# Immunotherapeutic Strategies Targeting Breast Cancer Stem Cells

**DOI:** 10.3390/curroncol31060232

**Published:** 2024-05-29

**Authors:** Maria Vasileiou, Sotirios Charalampos Diamantoudis, Christina Tsianava, Nam P. Nguyen

**Affiliations:** 1 Department of Pharmacy, School of Health Sciences, National and Kapodistrian University of Athens, 15771 Athens, Greece; mariavasileiou65@gmail.com; 2 Department of Pharmacy, School of Health Sciences, Aristotle University of Thessaloniki, 54124 Thessaloniki, Greece; s.c.diamantoudis@gmail.com; 3 Department of Pharmacy, School of Health Sciences, University of Patras, 26504 Patras, Greece; 4 Department of Radiation Oncology, Howard University, Washington, DC 20060, USA

**Keywords:** immunotherapy, breast cancer, cancer stem cells, immunological profile, CSC-targeted therapies, vaccines, CAR T cells

## Abstract

Breast cancer is the most commonly diagnosed cancer in women and is a leading cause of cancer death in women worldwide. Despite the implementation of multiple treatment options, including immunotherapy, breast cancer treatment remains a challenge. In this review, we aim to summarize present challenges in breast cancer immunotherapy and recent advancements in overcoming treatment resistance. We elaborate on the inhibition of signaling cascades, such as the Notch, Hedgehog, Hippo, and WNT signaling pathways, which regulate the self-renewal and differentiation of breast cancer stem cells and, consequently, disease progression and survival. Cancer stem cells represent a rare population of cancer cells, likely originating from non-malignant stem or progenitor cells, with the ability to evade immune surveillance and develop resistance to immunotherapeutic treatments. We also discuss the interactions between breast cancer stem cells and the immune system, including potential agents targeting breast cancer stem cell-associated signaling pathways, and provide an overview of the emerging approaches to breast cancer stem cell-targeted immunotherapy. Finally, we consider the development of breast cancer vaccines and adoptive cellular therapies, which train the immune system to recognize tumor-associated antigens, for eliciting T cell-mediated responses to target breast cancer stem cells.

## 1. Introduction

According to Global Cancer Observatory (GLOBOCAN) estimates for 2022, breast cancer (BC) is the second most prevalent cancer worldwide (11.6% overall), closely following lung cancer (12.4%) and the fourth lethal overall (6.9% of all cancer-related deaths) behind lung, colorectal, and liver malignancies [1]. BC is responsible for 2.26 million cases and 685,000 deaths globally in 2020 and is predicted to reach 30.19 million cases by 2040 [2]. Breast cancer can be divided into four subtypes based on specific biomarkers. The first type is human epidermal growth factor receptor 2 (HER2) overexpressing, accompanied by positive epidermal growth factor receptor (EGFR) status and negative estrogen receptor (ER) and progesterone receptor (PR) statuses (EGFR+, ER−, and PR−), followed by luminal A (ER+ and PR+, HER2−) and luminal B (ER+ and PR+, HER2+) types. Luminal A tumors typically express high levels of ER and PR, while luminal B tumors express high *ER* levels but reduced *PR* levels [3]. These tumors are the most heterogeneous and have the least prominent molecular drivers among the breast cancer subtypes. Lastly, there is triple-negative breast cancer (TNBC), which is characterized by the absence of *ER*, *PR*, and *HER2* [4]. Patients with TNBC had worse survival outcomes in every stage and sub-stage when compared to non-TNBC patients [5]. These different types of BC have distinct molecular and genetic characteristics, which can influence treatment options and prognosis. TNBC is considered to be the most immunogenic subtype and has drawn the attention of researchers who are conducting clinical trials to test immunotherapies in all subtypes of BC [6,7]. Triple-negative breast cancer (TNBC) has limited treatment options due to the absence of common drug target sites, making it challenging to develop effective therapies. Immune checkpoint blockade has shown promising results in TNBC treatment, with the Keynote 522 (NCT03036488) and Keynote 355 (NCT02819518) trials approving Pembrolizumab for early and metastatic stages, highlighting its efficacy in improving patient outcomes. Further research explores combinations of immune checkpoint inhibitors, such as Olaparib with Durvalumab or Atezolizumab (Atezolizumab was revoked due to unsatisfactory results for BC treatment according to the European Medicines Agency), Avelumab with Binimetinib, Sacituzumab Govitecan, or Liposomal Doxorubicin, and Pembrolizumab with chemotherapy, Binimetinib, or Sacituzumab Govitecan, with other therapies, highlighting the need for optimized treatment strategies [8,9,10,11]. The substantial inter- and intra-tumor heterogeneity that results in cell populations with variable sensitivity to therapies is a key barrier to treating BC. Breast cancer resistance to standard therapies is due to genetic, environmental, and cancer stem cell (CSC)-related factors. Immunotherapeutic approaches like cytotoxic T-lymphocyte-associated protein 4 (CTLA-4), programmed cell death-1/programmed cell death-ligand 1 (PD-1/PD-L1), chimeric antigen receptor (CAR) T cells, tumor antigens, vaccines, and targeted CSC therapies are promising treatments [12,13,14,15].

Immunotherapy is a novel cancer treatment strategy that employs the body’s immune system to recognize, target, and eliminate cancer cells. Immune checkpoint inhibitors, adoptive cell therapies (ACT), cancer vaccines, and cytokine therapy are used to boost the body’s natural defenses against malignant cells. The profound emphasis that trending therapies under development put on tumor-specific neoantigens in immunotherapy allows for personalized treatment regimens, fostering sustained immune responses against cancer recurrence and metastasis, and ensuring disease-free survival for a prolonged period of time [16,17]. While conventional therapeutic approaches, such as surgery, chemotherapy, radiotherapy, endocrinotherapy, and molecular targeted therapy, have shown significant improvements, particularly in breast cancer with targeted therapies like HER2 and ER, the spotlight seems to be on immunotherapies, considering the amount of potential that has been observed within the literature and the clinical setting. Traditional treatments may target specific tumor characteristics, leaving other subpopulations unaffected. In contrast, immunotherapy provides a platform upon which tumor-specific and more personalized approaches can be developed, paving the way for personalized treatment based on the patient’s immune profile [13]. Additionally, immunotherapy has demonstrated promising results in tackling the problem of tumor heterogeneity [18]. Current immunotherapeutic approaches can have fewer, yet significant, side effects since, through enhanced selectivity, they cause less harm to healthy tissues compared to chemotherapy, radiotherapy, and endocrine therapy. Immunotherapy has the ability to induce immunological memory, allowing the immune system to recognize and respond to cancer recurrence and metastasis, potentially leading to prolonged disease-free survival [13]. In fact, immunotherapy displays a synergistic effect when combined with chemotherapy agents [19]. Phase III of the IMpassion130 trial (NCT02425891) showed significant overall survival benefits in patients with PD-L1-positive TNBC patients who received atezolizumab in combination with chemotherapy [20]. Immunotherapy has also demonstrated durable responses, with some patients experiencing long-term remission even after discontinuing treatment, as demonstrated in the phase II study of pembrolizumab and capecitabine combination therapy for pretreated HER2-negative metastatic breast cancer (NCT03044730) [21].

Surgery is vital in the management of BC by removing the primary tumor and regional lymph nodes. It provides immediate tumor debulking and can effectively eliminate localized disease. However, surgery alone may not address the potential presence of residual CSCs or micrometastases that can lead to disease recurrence [14]. This appears to be of vital concern, given the significance of the role of CSCs in tumor aggression, which in turn is related to the size of their population, as Dhanota et al. observed utilizing the expression of aldehyde dehydrogenase 1A1 (ALDH1A1), one of the markers related to CSCs, along with the combination of ESA+/CD44 positive (CD44+)/CD24-negative or low (CD24−/low) and used in immunohistological methods such as fluorescence-activated cell sorting (FACS) [22], with Grade III (73.3%) tumors demonstrating elevated expression with Grade II (23.6%), which in turn showcased the same phenomenon when compared to Grade I (9.1%) [23]. By contrast, immunotherapy offers the potential for systemic and durable responses by activating the patient’s immune system to target cancer cells, including CSCs, both at the primary site and distant metastatic sites. The challenges lie in the identification and targeting of specific CSC markers and the development of immunotherapeutic strategies that can effectively eradicate these highly resistant and heterogeneous cells. Therefore, integrating surgery with immunotherapy may offer a comprehensive approach that combines the benefits of local tumor control with the potential for long-term immune-mediated tumor suppression. Comparing immunotherapy to radiotherapy for BC, it is evident that they have complementary roles and distinct mechanisms of action. Radiotherapy (RT) employs high-energy particles to destroy cancer cells within a specific treatment area. It is effective in eliminating tumor cells and reducing local recurrence [24]. However, radiotherapy primarily targets the bulk tumor mass and may not be as effective in eradicating CSCs, which are known to exhibit enhanced radioresistance. Immunotherapy has the potential to target CSCs and micrometastatic disease, as demonstrated by a meta-analysis of randomized trials in early TNBC. The addition of PD1/PD-L1 blockade to neoadjuvant chemo-immunotherapy significantly improves pathologic complete response rates (pCRs). This highlights the potential of immunotherapy to target CSCs and micrometastatic disease, offering a systemic approach that addresses both local and distant diseases [15].

Lastly, hormone therapy, also known as endocrine therapy, is primarily used for the treatment of tumors that are driven by estrogen or progesterone receptors [25]. While hormone therapy has proven to be highly effective in reducing the risk of recurrence and improving survival rates, it may not be effective for hormone receptor-negative BC, which does not rely on hormone signaling for growth. There is growing interest in exploring the combination of immunotherapy and hormone therapy. Preclinical and early clinical studies suggest that hormone therapy may boost the anti-tumor immune response and improve the efficacy of immunotherapy in hormone receptor-positive BC. For instance, hormonal therapies can modulate the tumor microenvironment (TME), making it more favorable for immune cell infiltration and activation. Additionally, hormone therapy-induced tumor cell death can release antigens that stimulate immune recognition and response [26,27,28]. Combination approaches that target both the hormone receptor signaling pathways and immune checkpoints hold promise for enhancing treatment outcomes and overcoming resistance mechanisms. Immunotherapy is a promising treatment for cancer and is currently being investigated as a potential stand-alone therapy or as a complementary treatment alongside conventional therapies such as chemotherapy and radiation. The ongoing research indicates that immunotherapy has the potential to provide significant benefits over traditional treatments, and the results so far have been encouraging.

With an extended lifespan that enables them to undergo multiple mutations required for oncogenic transformation, CSCs are a rare subpopulation of cancer cells that are presumably derived from stem cells. It is estimated that stem cells transform into cancer stem cells through the upregulation of existing stem cell pathways [18,29,30]. There have also been hypotheses that suggest that CSCs develop from progenitor cells, due to the more abundant nature of the tissue, or from differentiated cells through de-differentiation. All of these hypotheses dictate the subsequent self-renewal of proliferating cells, which is the foundation of dormancy [31,32,33,34].

**Figure 1:**
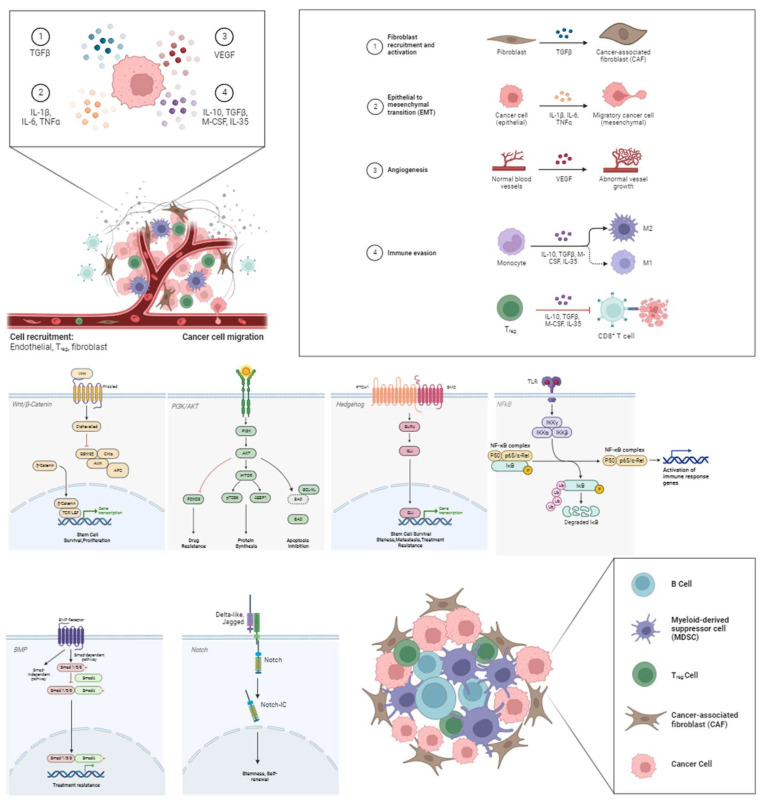
Influence of B-cells, myeloid-derived suppressor cells (MDSCs), regulatory T cells (Tregs) and cancer-associated fibroblasts (CAFs) on the TME through the Wnt/β-Catenin, PI3k/AKT, Hedgehog (HH), nuclear factor-kB (NF-κB), bone morphogenetic protein (BMP), and Notch pathway, respectively. CSCs release cytokines such as tumor necrosis factor-alpha (TNF-α), transforming growth factor-beta (TGF-β), interleukin 1β, 6, 10, and 35 (IL-1β, IL-6, IL-10, and IL-35), and vascular endothelial growth factor (VEGF), which regulate the tumor niche via the aforementioned corresponding intracellular downstream pathways following the uptake from the receptors. The result is the formation and the sustainment of the tumor microenvironment through angiogenesis, immune regulation, cancer cell proliferation, and fibroblast accumulation.

CSCs are responsible for the development, progression, and resistance of tumors to therapeutic treatments. The latter may be due to their capacity to suppress the immune system by hiding as dormant cells [35]. Tumor dormancy is a transitory phase that can last for years, up until an interaction with a tumor-permissive TME, which leads to exacerbation. CSCs release galectin-3, growth differentiation factor 15 (GDF-15), interleukin-10 (IL-10), interleukin-13 (IL-13), prostaglandin E2 (PGE2), and transforming growth factor-beta (TGF-β), which are known to modulate the tumor niche. Effective control over the TME hinges on mitigating pro-inflammatory cytokine secretion [36]. The TME is heavily influenced by various cell types such as regulatory T cells (Tregs), myeloid-derived suppressor cells (MDSCs), and B-cells, as shown in Figure 1. They function together with stromal cells, including fibroblasts or endothelial varieties, to support tumoral immunity and evasion patterns, thereby progressing the growth of cancerous cells. CSCs present an altered regulation capacity in many signal transduction pathways, such as PI3K/Akt, nuclear factor kappa B (NF-κB), Wnt/β-catenin, Hedgehog (HH), Notch, and bone morphogenetic proteins (BMPs) pathways [37,38,39,40,41,42,43]. These conglomerates play major stimulatory roles for attributes like migration, growth regulation, resistance to chemotherapy, radiotherapy, and progression toward epithelial–mesenchymal transition (EMT). Another integral finding dictates that microRNAs (miRNAs) play a central role in regulating stemness features, while controlling CSC-mediated tumorigenicity.

The Wnt/β-catenin pathway is frequently dysregulated in breast cancer stem cells (BCSCs), promoting self-renewal, proliferation, and EMT, thus contributing to tumor initiation and progression [43]. HH signaling, another pathway frequently activated in BCSCs, has been associated with the maintenance of stemness, metastasis, and therapy resistance in BC [38]. Notch signaling plays a role in maintaining the stem cell-like properties of CSCs, enhancing their self-renewal capacity, and influencing cell fate determination. Perturbations in BMP signaling can promote CSC expansion and contribute to therapy resistance [41]. The PI3K/Akt/NF-κB pathway is frequently hyperactivated in CSCs, promoting their survival, proliferation, and resistance to apoptosis [38,39].

Additionally, miRNAs play a significant role in regulating the stemness and tumorigenic properties of CSCs by targeting key components of these signaling pathways. Through interactions with mRNA targets, miRNAs can modulate the expression of genes involved in stem cell maintenance, differentiation, and tumorigenesis. For example, specific miRNAs can inhibit the Wnt/β-catenin pathway by targeting key components or activators of the pathway. Other miRNAs can regulate the activity of the hedgehog pathway by targeting its effectors or modulators. Similarly, miRNAs can modulate the expression of genes within the Notch, BMP, and PI3K/Akt/NF-κB pathways, thereby influencing CSC properties and tumor behavior [37,38,39,40,41,42,43].

Immunotherapy is greatly hampered by CSCs, which use immunosuppressive mechanisms and are resistant to standard therapies. Evasion and metastasis in BC are facilitated by a variety of cytokines, growth factors, and enzymes inside the intricate microenvironment. Attempts to target CSC populations are made more difficult by their variability and flexibility. Cancer stem cells also have distinct characteristics across different BC types, reflecting their biological behaviors and therapeutic responses. In triple-negative breast cancer (TNBC), CSCs are heterogeneous and interconvertible, displaying unique responses to chemotherapy. Mesenchymal CSCs (M) have CD44+/CD24− and elevated levels of Yes-associated protein (YAP), nuclear factor kappa-light-chain-enhancer of activated B cells (NF-κB), and enhanced metabolic pathways. Epithelial CSCs (E) have ALDH+, heightened Wnt signaling, NF-κB activity, hypoxia response, and enhanced glutathione metabolism. Hybrid E/M CSCs in TNBC are more tumorigenic than their purely epithelial or mesenchymal counterparts and can differentiate into either cell type [44,45]. In HER2-positive breast cancer, CSCs are characterized by the CD44 high/CD24 low phenotype and ALDH1 expression. These CSCs are associated with resistance to anti-HER2 agents, such as trastuzumab, and are more frequently detected in recurrent breast cancer compared to primary tumors [46,47]. In luminal-type breast cancer, CSCs are identified by specific surface markers, such as CD44+/CD24−/low cells. The proportion of CD44+/CD24−/Hoechst-cells correlates positively with sphere-forming ability, indicating strong stemness properties. These CSCs overexpress stemness-related genes, including OCT4, NANOG, and KLF4, which are crucial for maintaining their self-renewal potential and pluripotency. Additionally, luminal-type breast cancer exhibits heterogeneity within its CSC population, characterized by the presence of both estrogen receptor (ER)-positive and ER-negative cells [48].

The categorization of breast cancer stem cells (BCSCs) into different subtypes, based on specific markers and characteristics, provides valuable insights into the heterogeneity of breast cancer. Luminal-type BCSCs are associated with luminal-type breast cancer and may exhibit markers indicative of luminal cell properties. Triple-negative BCSCs, lacking expression of estrogen receptor (ER), progesterone receptor (PR), and human epidermal growth factor receptor 2 (HER2), represent a distinct subtype with unique characteristics. Hormone receptor-positive BCSCs express ER and/or PR, aligning them with hormone receptor-positive breast cancer. HER2-amplified BCSCs, characterized by the amplification of the HER2 gene, form another distinct category. Since distinct BCSC subtypes may react differently to medicines targeting particular markers or pathways, understanding these subtypes is essential for customized treatment approaches. This classification emphasizes the significance of individualized therapies based on subtype-specific features and underscores the complexity of BCSCs within breast cancer. This literature review aims to provide further details on immunotherapeutic tactics, emphasizing defining particular CSC indicators, comprehending immune evasion mechanisms, and proposing innovative approaches to eliminate BCSCs effectively [49].

## 2. CSC Immune Checkpoint

### 2.1. Immune Checkpoint Molecules

The expression variability of immune checkpoint molecules in BCSCs plays a detrimental role in immunity evasion, metastasis, and treatment resistance. In fact, checkpoint molecules can undergo alterations such as the downregulation of MHC class I polypeptide-related sequence A/B (MICA/B) ligands—which pose as a precondition for physiological natural killer (NK) cell stimulation—through the natural killer group 2 member D (NKG2D) receptor, or overexpression of CD47 and PD-L1 through the hypoxia inducible factor (HIF) and EMT/β-catenin/STT3/PD-L1 pathways, respectively. These altered molecules find potential in detecting, characterizing, and combating BCSCs. Detection usually occurs through proteomic analysis methods and genome comparison through next generation sequencing (NGS).

The functionality of these molecules can be translated into real-world clinical applications through the utilization of immune checkpoint inhibitors. However, for such an intervention, for example the ones targeting PD-1/PD-L1, to be clinically applicable and a better alternative to conventional approaches, cancer cells need to be positive for the corresponding molecules. There have been findings that indicate a positive correlation between PD-L1 expression and stemness of the tumor, through markers such as ALDH1A1 and OCT3/4, thus enhancing stemness along with metastatic potential and ability to evade immune responses [50,51]. Regarding the occurrence of PD-L1/PD-L2 in CSCs of the MCF-7 BC cell line, when contrasted against parental MCF-7 cells, cellular PD-L1 protein is remarkably increased in sphere-forming cells. As such, there is evidence that PD-L1 expression is increased in CSCs, which poses as a promising factor for PD-1/PD-L1-based immunotherapy with the aim of attaining robust and long-lasting favorable results [52].

However, studies have demonstrated that the probability of success of the immune checkpoint blockade in a clinical setting is correlated to the level and the number of mutations present in the tumor cell population [17,53,54]. In addition, the reported lack of clinical response is derived from the lack of human leukocyte antigen (HLA) class I expression observed in tumor cells [55,56]. Therefore, based on the lack of evident ability of CSCs to cause adequate immune response, the option to follow the path of the checkpoint inhibitors could fail to cause extermination of these cells.

### 2.2. Dendritic Cells (DCs)

It is well established that dendritic cells (DCs) have a crucial role in commencing innate and/or adaptive responses of the immune system through their antigen-presenting capabilities [57,58,59,60]. When it comes to CSCs, DCs capture and present tumor-related antigens to other populations of immune cells, which are stimulated by cytokines excreted by the former [61].

TGF-β is an important molecule, since it promotes cancer development in the later stages [62,63]. The result of its interaction is reported to be the reduction in DC activation and capabilities, as it halts the production of the costimulatory molecules CD80 and CD86 and activates the Wnt/catenin that stands as an obstacle to the stimulation of basic leucine zipper transcription factor ATF-like 3 positive (BATF3+) DC cells and renders the tumor resistant towards anti-PD1 treatment courses, despite the enhancement of PD-L1 expression. Additionally, DCs release C-X-C motif chemokine ligand 12 (CXCL12), which supports CSCs stemness [61].

Investigating therapeutic approaches, it is recorded that DCs can be implemented in some proposals. The vaccination with antigens related to and/or expressed on CSCs, such as aldehyde dehydrogenases 1 (ALDH1A1) and 3 (ALDH1A3), CD44, and epithelial cell adhesion molecule (EpCAM), seems to be under the spotlight [64]. In this way, the response by cytotoxic T-lymphocytes (CTLs) is enhanced and more efficient. However, something that should not in any case be overlooked when opting for tumor markers is the affinity that they bind with major histocompatibility complex (MHC) molecules, as this can be proven as a determining factor in productive antigen presentation [65]. In addition to peptides, RNA fragments can also be used as antigens [66].

### 2.3. Tumor-Associated Macrophages (TAMs)

The dynamic relationship between CSCs and tumor-associated macrophages (TAMs) is an incredibly important factor in the progress of the disease. When it comes to BC, it has been observed that CSCs lead to monocyte activation via the expression of CCL2 polarization to M2, the most prevalent population amongst TAMs. This phenomenon can be easily characterized by the enhanced ALDH1 activity, as well as the presence of sex-determining region Y-box 2 (SOX-2), Nanog, and octamer-binding transcription factor 3/4 (OCT3/4) [67]. The recruitment of peripheral macrophages to the TME is usually triggered by the excretion of chemokines such as members of the groups CCL, CXC, and interleukins (IL), as well as colony-stimulating factor (CSF) 1. Specifically regarding BC, CXCL1, CXCL 12, CCL2 and IL-6 have been reported to have significant roles in the recruitment of macrophages, and therefore pose as significant potential targets for immunotherapeutic solutions [68].

Epidermal growth factor (EGF), which has a crucial role in tumor invasiveness, is produced, in part, by TAMs. TAMs are stimulated through the excretion of M-CSF/CSF-1 by the CSCs [64]. Additionally, studies have demonstrated that TAMs possess the capability of controlling the “stemness” properties of the tumor sites, through the production of a number of cytokines, such as some of the ones mentioned above. Also, by activating the signal transducers and activators of transcription 3 (STAT3), TAMs play a significant role in assisting the survival and multiplication of CSCs [66,67]. Besides the aforementioned, TAMs can also influence the phenotype and operational effectiveness of antigen-specific T cells [63].

In addition, it has been observed that BC cells are able to cause the production of IL-6 via the macrophages located in the TME, through p-38 activated protein-1 (AP-1)-dependent mechanisms [65]. Complimentary to the above stands the fact that production of IL-6, IL-18, and granulocyte–macrophage colony-stimulating factor (GM-CSF) is stimulated by the binding of TAM’s CD90/Thy-1 with CSCs’ ephrin-A receptor 4 (EphA4) [69]. Thus, through the mediation of TAM-produced IL-6, the phenotype of the CSCs is enriched, along with enhanced stemness, antigenicity, migration ability, and growth, as well as immune evasion [65,66]. The amount of evidence suggests that TAMs are worthy of investigation as a target for the treatment of BC.

### 2.4. NK Ligands

Strong indications suggest that NKs are able to effectively and efficiently target CSCs through the mediation and subsequent activation of cytokines. For this to occur, NK cells rely on a number of ligands and receptors, located on the cellular membrane of tumor cells. Regarding CSCs, the method of recognition is independent from HLA class I, due to the lack of such molecules, and relies on other receptors, such as natural killer protein 30 and 44 (NKp30, NKp44) and UL16-binding protein 1 and 2 (ULBP1, ULBP2) [70]. In fact, an upregulation has been observed in the production of stress-related NKG2D ligands, as well as death receptors DR5 and Fas [71,72]. Additionally, it has been observed that NK cells use both granule-mediated and death receptor mechanisms to induce apoptosis and that the cytotoxicity increases with the ratio of NK to target MDS-MB-231 [70]. According to ex vivo studies in mouse models, when NK cells were activated and infused, neoplasms seemed to be of a smaller size, when compared to the controls [71].

However, there seems to be a plethora of factors that contribute to immune evasion through the aforementioned mechanisms, leading to treatment resistance and the increased risk of metastasis [73]. These factors include the ability of NK cells to relocate towards the site of the neoplasm, identify, and kill the target. In a study comparing NK cytotoxicity in MDS-MB-231 vs. CD24−/low/CD44+, it was found that evasion is induced through the upregulation of HLA class I, E, and G molecules, which have an inhibitory effect on NKs and CTLs and promote the downregulation of MHC class I chain-related molecules A/B, which induce cytotoxic activity against the target.

These findings imply that NK cells can be implemented in clinical practice to target CSCs through a variety of therapeutic approaches. Notable methods include the enhancement of the activity of autologous NK-like cytokines induction into cytokine-induced killer (CIK) cells or the infusion of engineered CAR-NK cells carrying receptors targeting CSC antigens, such as chondroitin sulphate proteoglycan 4 (CSPG4), epidermal growth factor receptor (EGFR), and CD44v6 [72].

### 2.5. Cytokines

Cytokines are molecules that function as signals in the communication between cells. Therefore, it can be safely assumed that they have an important role in the progress, properties, and eventual fate of neoplasms. A primary example that contributes to the enhancement of the neoplasm would be the altering of non-CSCs to CSCs, under the influence of cytokines, emitted by the stromal cells [74]. However, cytokines work bilaterally, as CSCs use them in order to evade immune response and develop tolerance, but also to induce differentiation through molecules, such as IL-10 and IL-13. Considering the effect that interferon alpha (IFN-α) has on the activation of the immune system, multiple IFN-α therapies can be proposed and implemented [75,76].

Cytokines have a potential pro-tumorigenic and anti-tumorigenic effect and operate as a recurrence indicator. IL-1 can be divided into two activating cytokines, IL-1α and IL-1β. The former is associated with dedifferentiation and lymphangiogenesis, which is promoted by the expression of HER2, encouraging the generation and retention of CSCs. The latter is shown to be present in breast tumors, leading to production of growth factors, such as EGF and TGF-β, which are correlated with a relatively worse prognosis [76]. Additionally, IL-6 encourages angiogenesis, metastasis, and BCSCs’ self-renewal, from non-stem cancer cells (NSCCs) to CSCs [77]. The response to IL-6 is variable and depends on factors such as the concentration, number of receptors, and NSCCs to CSCs ratio [60]. Specifically, IL-6 enhances BCSC production, through the STAT3-mediated NF-κB transcription activation, which results in the production of proinflammatory cytokines and a positive feedback loop, confirming the assumption that BCSCs are IL-6 dependent for their survival, plasticity, and spread [76,77]. Finally, IL-8 is present in high levels in CSCs, with elevated ALDH activity, while its concentration is linked to the span of the population [76].

TGF-β is a cytokine, present in elevated levels in the TME, that regulates the growth and activity of BCSCs [78]. According to transcriptomic analyses, there has been an elevated TGF-β expression observed in BCSCs, which implies that the TGF-β pathway may be the answer to drug resistance and recurrence [77]. This factor also has a notable effect on immune cells, as it suppresses T cells via the forkhead box protein 3 (Foxp3) stimulation and acts against the infiltration of NK cells [76].

## 3. CSC Immune Evasion

Tumor dormancy has been defined as the possession of the capability of evasion from immune surveillance, as well as passive dwelling within tissues by cancer cells, while causing tumor development and metastasis, up until they interact with the TME [79,80,81,82]. A TME consists mainly of two types of cells, immune and stromal ones. The population of immune cells is diverse as it includes tumor-associated macrophages (TAMs), tumor-infiltrating lymphocytes (TILs), regulatory T cells (Tregs), myeloid-derived suppressor cells (MDSCs), dendritic cells (DCs), and NK T cells. Stromal cells, also known as mesenchymal stem cells (MSCs), include blood endothelial cells (BECs), lymphatic endothelial cells (LECs), fibroblasts, and pericytes. Another type of immune cell that evidently supports the CSCs are the MDSCs, as they promote microRNA-101 expression and, thus, the expression of stemness genes, while suppressing other immune cells in the TME [83,84,85,86,87,88].

The interaction between CSCs and the TME can be divided into two types: the contact-dependent mechanisms that rely on the interaction of CSCs with another cell or with the extracellular matrix (ECM), and contact-independent mechanisms that utilize molecules, such as cytokines and growth factors. Regarding contact-dependent mechanisms, a number of studies indicate that CSCs are able to evade immune response by dampening the function of DCs. This can be achieved through a reduction in DCs in the TME, crippling their maturation and differentiation through the expression of alpha fetoprotein, the emission of immunosuppressive cytokines, such as IL-10, IL-4, and IL-13, and the expression of inhibitory molecules, such as indoleamine 2, 3-dioxygenase (IDO). IDO converts tryptophan to kynurenine, which, in turn, triggers the transformation of naive T cells into Tregs. These are immunosuppressive cells that inhibit the antitumorigenic function of several immune cells, including macrophages, lymphocytes, natural killer NK cells, and dendritic cells. Under physiological conditions, these cells recognize microbial pathogen-associated molecular patterns (PAMPs) or damage-associated molecular patterns (DAMPs), and act accordingly via the production of immune sensitizing molecules, initiating an immune response [89]. In fact, chemokine receptor CXCL12, which is produced by the TME, can play a crucial role in the in situ recruitment of Tregs, MDSCs, and DCs, contributing to CSC self-renewal [90].

A major mechanism, among numerous cancer types, through which CSC immune evasion is achieved, involves relatively lowered antigen processing and presentation, which, by itself, is a significant factor in the decrease in antigenicity. These include transporters associated with antigen processing (TAP) and/or MHC molecules. TAP is detrimental for the internal transportation of peptide molecules from the cytosol that are bound to the endoplasmic reticulum, where they undergo processing by MHC complexes and subsequently participate in the antigen presentation on the cell surface, which is crucial for antigen surveillance by T cells. Therefore, the downregulation of TAP and MHC molecule expression grants the means of T cell evasion to CSCs [91]. Interestingly, while an increased susceptibility to attack NK cells due to decreased MHC class I (MHC I) molecules would be expected, the opposite effect is observed, with the decreased expression of NK cells and NK cell-mediated cytotoxicity. The NK cell signaling pathway is typically induced by the binding of NK immunoglobulin-like receptors to MHC I molecules [92,93,94,95].

It is important to note that the inflammatory environment seems to affect the interaction between CSCs and the TME. When the inflammation fails to be resolved, the secretion of cytokines as well as EGF, TGF-β, and fibroblast growth factors, followed by the secretion of proteolytic enzymes, give rise to cancer cells as they regulate the ECM detachment [89]. Prolonged ECM detachment, also known as metastatic dissemination, creates an environment with a relatively high concentration of ROS and the inhibition of fatty acid oxidation (FAO), which results in an energy crisis within the cancer cell and engagement of pathways associated with cell death [92].

Contact-independent mechanisms also play a significant role in CSC immune evasion through a positive feedback loop, which is facilitated via the production of cytokines, such as IL-6, TNF, and TGF-β. These cytokines can kickstart HH signaling in CSCs [96]. The HH pathway interacts with other tumorigenic signaling pathways that include, besides others, NF-κB, mitogen-activated protein kinase (MAPK), phosphatidylinositol 3-kinase (PI3K), and epidermal growth factor receptor (EGFR) cascades [97,98,99]. Through the aforementioned mechanisms, DCs are capable of promoting chemo-resistance and tumorigenicity, as observed in follicular lymphoma CSCs [100,101]. Another contact-independent mechanism involves the upregulated expression of death molecules, primarily through the Fas ligand (FasL) and the tumor necrosis factor (TNF)-related apoptosis-inducing ligand (TRAIL). FasL manages to establish an immune barrier within the endothelium, enabling cells to trigger Fas-mediated apoptosis on CTLs, but not Tregs. Tregs present with an increased presence of cellular FLICE (FADD-like IL-1β-converting enzyme)-inhibitory protein (c-FLIP) on the cellular membrane, making them resistant to FasL exposure [102]. In addition, CSCs are defined by the increased expression of the immune-checkpoint protein PD-L1, whose action is to mainly alter T cell function with the purpose of inhibition through binding to its cognate receptor, the programmed cell surface expression of PD-L1 [89]. PD-L1 production in CSCs is directly regulated by STAT3, which also promotes an immunosuppressive TME and the expansion of MDSCs and Tregs [93,94]. This, in turn, has a significant role in promoting the production of OCT-4A and Nanog transcription factors, which sustain the stemness of BC via the activation of the PI3K/AKT pathway [95]. Further studies are required to interpret the immunologic profile of CSCs and the communication between them and the TME, in order to fully comprehend the concept of immune evasion.

The aforementioned mechanisms may differ among BCSCs types. These include the epithelial state characterized by high ALDH activity, and the mesenchymal state characterized by high CD44 and low CD24 activity. The conversion between the epithelial and mesenchymal states is a typical characteristic of BCSCs, which allows them plasticity. The epithelial state is typically located centrally, while the mesenchymal state is located at the front of the tumor. Recent studies report the highest proportion of epithelial BCSCs and ALDH activity in HER+ BC with a poor prognosis. In addition to high ALDH expression, CD44+/CD24− expression seems to contribute to a poor prognosis. This hypothesis is supported by in vivo findings in radioresistant MCF7 BCSCs, where HER2+ CD44+/CD24− BCSCs displayed aggressive tumorigenesis, compared to HER2− CD44+/CD24− BCSCs. However, a larger number of studies in HER2+ BCSCs is required to fully establish this hypothesis [46].

## 4. Immunotherapeutic Strategies

Interventions involving immunotherapeutic approaches aim to target CSCs utilizing immune cells such as CIK cells, NK cells, CD8+ T cells, and γδ T cells, with the latter being ideal BCSC targets [103,104]. An additional method of targeting CSCs would be DC-based vaccines. There have been multiple applications of immunotherapy implemented, including the following: adoptive T cell therapy, which involves TIL isolation, culturing, and reinfusion back to the patient; oncolytic virotherapy, which, via immunogenic cell death and the activation of T cells, aims to induce antitumor immunity [105]; DC-based vaccines, which target BCSCs [106]; and combinations with other immunotherapies. Table 1 provides a summary of immunotherapeutic strategies, their intervention and therapeutic effect. According to recent clinical data, the majority of immunotherapeutic interventions implement a combined path of therapy, which is quite often includes oncolytic viruses, DC-based vaccines, and immune checkpoint blockades.

### 4.1. Adoptive Cell Therapy (ACT)

The basic principle of adoptive cell therapy (ACT) involves the alternation of immune cells located on the tumor site or circulating the body [107,108]. There are three types of ACTs, including TILs, TCR, and CAR-T cell, with the latter demonstrating promising results in terms of BCSC identification and targeting [109,110].

Antigens that occur only after tumor-specific somatic mutations, also known as neoantigens, can be utilized for the ex vivo formation of T cells which target neo-antigen-expressing CSCs [111]. The specificity and efficacy of said immunotherapy can be ensured through the targeting of a specific CSC neoantigen related to BC. A number of neoantigens are currently being investigated in pre-clinical models and have demonstrated significant amounts of potential. In vitro experiments in U-251 MG, T98G, U-87 MG, and HTB185 glioblastoma cell lines showed that NKG2D CAR-T cells effectively targeted cancer stem cells, as confirmed by the elevated production of NKG2D ligands [112]. NKG2D expressing CARs have shown positive results when it comes to effectiveness against most cancer types, such as breast cancer, as well as lung, colon, ovarian carcinoma and glioma, neuroblastoma, leukemia, and melanoma [113,114,115,116,117,118]. A strong connection has been found, within the aforementioned cancer types, between the occurrence of soluble MICA in the serum and the level of limitation in the rate of expression of NKG2D on tumor-infiltrating and peripheral CD8+ T cells. However, there are multiple parameters affecting the regulation of serum NKG2D ligand expression, independent of soluble MICA [116].

Moreover, CD90, CD49, CD44, CD24, and ALDH, in addition to EpCAM, are a few surface markers reported for the isolation of BCSCs, as they tend to have altered expression levels when compared to those of other bulk tumor cells [119,120,121]. CD90, also known as Thy-1, is a membrane GPI-anchored glycoprotein (25–35 KDa), expressed on various stem cells with an important effect on inflammation, cell adhesion, and stem cell differentiation [122,123,124]. CD90 is mainly expressed in the leukocytes, bone marrow-derived mesenchymal stem cells, and hepatic stem/progenitor cells (HSPCs) [125,126,127,128], and has been identified in murine BCSCs [129]. The role of CD90 has been highlighted in HCC cell lines where CD90+ cells possessed more tumorigenic properties than CD90- cells [123]. In addition, the role of CD44 and CD24 markers has been extensively studied in CD44+/CD24− cells, which are indicative of a poor prognosis. Al-Hajj et al. (2003) isolated CD44+/CD24− cells from BC tumors which had an enhanced ability to produce tumors in immunodeficient mice, serving as targets to eliminate the BCSC population [130,131]. According to Ginestier et al. (2007), the expression of ALDH, a cluster of enzymatic molecules that have an active role in the metabolism of aldehyde derivatives, was utilized as a marker of unfavorable results in BC patients, as it was correlated with enhanced oncogenic capabilities and resistance to antineoplastic molecules. In vitro studies in HLA-A2+ breast cancer cell lines noted that the elimination of CSCs with ALDH-specific CD8+ T cells, severely limited the development of the tumor as well as metastases, while in vitro studies in xenograft-bearing immunodeficient rodents reported prolonged survival [132,133].

EpCAM is another surface marker and tumor-associated antigen (TAA), more widely expressed on CSCs than initially reported, but not limited to CSCs [134,135]. In fact, the increase in EpCAM expression has been noted to be as high as 100–1000 times, when compared to normal levels in primary and metastatic BC [136]. This phenomenon concerning primary breast cancers is related to unfavorable disease-free and overall survival, independent of tumor size, nodal status, histological grade, and hormone receptor expression [137]. Al-Hajj et al. (2003) demonstrated that the EpCAM+, CD44+, CD24−, and lineage− fraction of BCSC had a >10-fold increase in the frequency of tumor-initiating cells, when compared to the EpCAM−, CD44+, CD24−, and lineage− BCSC fraction. Ongoing clinical trials are investigating new-generation CAR-T cells targeting CD44v6 (NCT04430595) and EpCAM (NCT02915445) surface antigens as a safe, feasible, and effective intervention for advanced BC [138,139].

These data shed light on the diagnostic and therapeutic potential of EpCAM and cater to their value as a candidate target for CAR-T cell therapy. A representative example would be the clinical evidence, which has demonstrated that up to 30% of breast cancer patients have a higher chance of developing bone marrow micrometastatic disease at the time of diagnosis. However, after 5 years, only 50% of said patients will present with clinically evident metastases, which can be attributed to tumor dormancy. This could be alternatively attributed to carcinogenesis through the spread of nontumorigenic and tumorigenic cancer cells [131]. The identification of BCSCs markers also finds therapeutic applications in overcoming treatment resistance, due to the failed targeting of tumorigenic cells, resulting in the recurrence of the metastatic disease. It is evident that stem cells have mechanisms that act catalytically in the development of resistance to chemotherapeutic interventions, such as an increased expression of membrane transporters (for example, the breast cancer drug resistance protein). Thus, the identification of BCSCs surface markers provides the ability to differentiate and target cell populations, in order to overcome treatment resistance and implement more effective therapies [131].

### 4.2. Oncolytic Virotherapy (OVT)

Oncolytic virotherapy (OVT) poses itself as a relatively new and developing solution in the field of cancer therapeutics, often used in combination with other immunotherapies. OVT utilizes oncolytic viruses that specifically target and eventually lyse cancer cells, avoiding those ones of the physiological tissue. The resulting immunogenic cell death induces antitumor characteristics, via the activation of the T cells [140]. The nature of OVs as a targeted approach for particular types of cancer cells has facilitated their therapeutic use as a nanomedicine in OVT [141]. Interestingly, the overexpression of the receptors for intercellular adhesion molecule 1 (ICAM-1) and decay-accelerating factor (DAF) caters to the effective entry of coxsackievirus into breast cancer cells. Experimental evidence indicates that, despite the cooperation between ICAM-1 and DAF in viral binding, the expression of ICAM-1 is necessary for the completion of a successful lytic infection [142]. In addition, Gholami et al. (2012) examined the antitumor properties of GLV-1h153, a Lister strain, in both in vivo and in vitro models of TNBC. After the passage of five weeks of treatment, all samples of lymph nodes and organs had demonstrated a complete response to GLV-1h153 treatment with no evidence of metastasis. It is worth mentioning that this viral therapy holds the advantage of carrying the human sodium iodide symporter gene. Human sodium iodide symporter is a membrane transporter protein normally detected on cells and is responsible for the uptake of radioactive material, such as Tc-99 and iodine (I-124, I-131). Therefore, it can be a valuable element in the detection of breast tumors via positron emission tomographic imaging [143]. An intervention that is currently being investigated is combination therapy with the vvDD strain. A phase I intratumoral dose escalation clinical trial of vvDD was conducted in 16 patients with advanced BC to determine its safety, clinical response, pharmacokinetics, and pharmacodynamics, as well as signs on the viral life cycle and the infection of distant tumors. While there was evidence of a response, there was no true clinical benefit achieved [144].

Similar efforts have been made to assess whether the VACV GLV-1h68 strain can eliminate CSCs that have developed resistance to irradiation and chemotherapy. In vitro studies in human breast cell lines (MCF-7, MDA-MB-231, and HS578T) have demonstrated the result that the strain replicated more efficiently in cells with higher ALDH1 activity that possessed similar qualities to stem cells, compared to those with lower ALDH1 activity. Therefore, the GLV-1h68 strain serves as a potential agent against stem-like cells or BCSCs, both in primary and treatment resistant metastatic breast cancer [145].

### 4.3. DC-Based Vaccines

To date, the most-studied immunotherapy approach involves the promotion of tumor cell recognition and eradication by the cells of the immune system via the utilization of DC vaccines, whereby BCSCs are seen as antigen sources for the initiation of a tumor-specific immune response [146,147]. There are a number of ongoing or completed clinical trials that look into the effects of CSC-enriched populations on different syngeneic immunocompetent hosts. The results demonstrate that in vitro BCSC-DCs were capable of significantly inhibiting BCSC proliferation at a DC:CTL ratio of 1:40, when inserted into the circulation of BCSC tumor-bearing rodents. In fact, the inhibition of BCSC-DCs on BCSC proliferation seems to be variable, as different ratios of DCs:CTLs have been recorded, with the most significant inhibition occurring at an ideal ratio of 1:40. This can also be confirmed with the change in tumor size, with a decrease by 23% in the BCSC-DC groups, versus an increase by 14% in the control group. Therefore, this stands as evidence of significance in indicating the effectiveness of therapeutic treatment with DCs primed by BCSC-derived antigens [148]. Furthermore, in vivo studies in nonobese diabetic/severe combined immunodeficiency (NOD/SCID) mouse models showed that the BCSC-primed DCs prolonged the survival time by 70%, while 10% of mice were still alive after 120 days of treatment, followed by monitoring [149]. Positive results were also recorded in oncologic patients with metastatic breast adenocarcinoma, injected with lysate-pulsed DCs of ALDEFLUOR-positive cells (NCT02063893). The inhibition of CSCs by CTLs generated from peripheral blood mononuclear cells or splenocytes harvested from CSC-vaccinated hosts was recorded under in vitro conditions [150]. Overall, DC-based vaccines have demonstrated their effectiveness both in vivo and in vitro, inhibiting tumor growth in a specific manner. These findings suggest the use of DC vaccines as a viable option for the targeted inhibition of BCSCs, as well as cancer stem cells in general.

### 4.4. Other Immunotherapeutic Approaches

#### 4.4.1. NK Cells

Other than ACT-, OVT- and DC-based vaccines, other immunotherapeutic approaches are also being explored to target and eliminate BCSCs. Increasing amounts of evidence suggest that the identification and lysis of CSCs can selectively occur by the NK cells in solid tumors, focusing on the condition of combination therapy that simultaneously targets non-CSCs and CSCs. Although NK cell immunotherapy has significant shortcomings in the treatment of advanced solid tumors when used as monotherapy, it effectively targets CSCs with diverse solid tumor type origins, a condition that is signified when combined with RT [151].

Yin et al. (2016) noted that CD44+CD24− CSCs were vulnerable to the interference of NK, suggesting that NK cells might have a targeted effect on cancer stem cells. They specifically reported that BCSCs demonstrated enhanced sensitivity to NK cells activated by IL-2 and IL-15, mediated by high levels of production of the NKG2D ligands ULBP1, ULBP2, and MICA on CD44+, CD24− human breast CSCs. It has been noted that the expression levels of said ligands were remarkably higher on CD44+CD24− CSCs, thus leading to the conclusion of potentiated recognition by NK cells. A cytotoxic assay demonstrated that there was no dependence on NKG2D for the enhanced sensitivity of CSCs to NK cells to occur. Besides BCSCs, it was also noted that human CD133+ colon cancer stem cells displayed sensitivity to NK cells’ cytotoxicity as well. These aforementioned phenomena contribute to the suggestion that CSCs possess sensitivity to NK cells mediated by NKG2D, as they facilitate the interaction with key ligands [152]. Factors of high importance for the NK targeting of CSCs involve pre-treatment and post-radiation treatment, in order to eliminate non-CSCs and CSCs, respectively. Taking into consideration the expansion of the CSC population post-radiation and immediately after surgical resection, it is safe to speculate that NK cell immunotherapy is of clinical significance. This hypothesis can be confirmed with in vivo results from tumor-bearing mice with local RT that received pretreatment with NK prior to radiation exposure. Despite the potential of a combined immunotherapy and radiotherapy approach aimed against CSCs and non-CSCs, the consideration of several parameters is paramount for proper treatment implementation. Future studies should investigate the optimal post-radiation treatment window, in order to determine the maximum NK efficacy. It is suggested that NK cells are administered in a short period of time post-radiation, with a maximum gap of 1 week between RT exposure and NK cell transfer [153].

#### 4.4.2. Mesenchymal Stem Cells (MSCs)

Mesenchymal stem cells (MSCs) belong to a category of stem cells received from allogeneic murine bone marrow, specifically from immature DCs, which are primed with MSC-derived antigens. Similarly to BCSC-DCs, MSC-DCs have potential for eradicating BCSCs, when administered into the circulation of BCSC tumor-bearing mice. However, when comparing MSC-DCs to BCSC-DCs, BCSC proliferation was not significantly affected by the activity of MSC-DCs, which led to 47% increase in tumor size. The failure to reduce the breast tumor mass signifies a lack of immune response, which did not take place in the group of mice that were treated with MSC-DCs, as opposed to BCSC-DCs. It remains unclear as to why intravenous treatment with MSC-DCs led to an increase in tumor mass. The latter is most likely attributed to the phenomenon of a relatively weak immune response against the neoplasm, as MSC-DCs most likely induce a large number of immune cells specific to MSC, thus facilitating tumor growth. It is our consideration that further investigation in cell lines with DCs primed with MSC-derived antigens is needed to gain more clarity regarding this finding [148].

#### 4.4.3. Signaling Regulation

Hedgehog signaling, a pathway frequently activated in BCSCs, has been associated with the maintenance of stemness, metastasis, and therapy resistance in BC [80]. Notch signaling pathways are important factors in maintaining the properties of CSCs that are attributed to stem cells, such as enhancing their self-renewal capacity, as well as influencing the onset and progression of many cancer types, including breast cancer, as many of its substrates are highly implicated in disease pathogenesis and malignancy [154,155]. The release of the intracellular domain of Notch receptors, mediated by the γ-secretase enzyme, is paramount for the activation of the Notch signaling pathway. Therefore, the antagonistic targeting of γ-secretase, alone or when combined with other therapies, may inhibit the Notch pathway and tumor progression. Moreover, the PI3K/Akt/NF-κB pathway is frequently hyperactivated in CSCs, promoting their survival, proliferation, and resistance to apoptosis [76,77]. The PI3K/Akt/NF-κB pathway has shown positive outcomes in BCSC-based therapies, especially for HER-2 positive cancers, with researchers reporting reduced tumor growth and metastasis, which result in improved patient outcomes [156].

#### 4.4.4. Metabolism Regulation

The unique metabolism of BCSCs serves as an alternative approach to BCSC-specific treatments. Specifically, the metabolism of BCSCs includes aerobic glycolysis and OXPHOS, which are prevalent in BCSCs, as well as fatty acid oxidation and iron metabolism. The B-cell lymphoma 2 (BCL2) protein, along with the peroxisome proliferator-activated receptor γ inhibitors, have the ability to limit the rate of oxidative phosphorylation and minimize BCSC growth and metastasis [157,158]. Contemporary results have highlighted the significance of FAO and iron metabolism in CSCs and suggest that specific targeting can be utilized to suppress BCSC growth and proliferation. It is known that CSCs have a relatively heavy reliance on the activity of elements of the lipid metabolism, including stearoyl CoA desaturase 1 (SCD1) and hydroxymethylglutaryl-CoA (HMG-CoAR) which are associated with the Hippo and Wnt pathways. The uncoupling of FAO from ATP synthesis results in excessive lipid build-up in cytoplasmic organelles, in the form of LDs. Recent observations suggest that there is a strong connection between LD content, the synthesis of CD133, and the activation of Wnt signaling. Lastly, iron uptake has been found to be enhanced in BCSCs, followed by low divalent metal transporter 1 (DMT1) levels and the overexpression of iron regulatory protein 2 (IRP2), a key regulator of the production of iron-related proteins at a translational level. However, discrepancies concerning adherence to standards on CSC isolation must be taken into consideration, to avoid the wrong interpretation of the interplay between CSCs and their metabolic status [159,160,161].

## 5. Limitations and Challenges

### 5.1. Adoptive Cell Therapy (ACT)

Despite the standard utilization of adoptive cell therapy (ACT), there are a number of challenges that ought to be overcome. Initially, the main concern regarding CAR T cells being used against BCSCs is the occurrence of adverse effects, which are mainly caused by non-targeted CAR T- cell activity, as BCSC surface markers overlap with the ones presenting on physiological cells. There have been multiple reports regarding ACT-mediated toxicity, with adverse reactions (AEs) ranging from B-cell aplasia to acute respiratory distress syndrome, which is a life-threatening condition [162,163]. An additional obstacle would be the heterogeneity between various CSCs, with CSCs expressing multiple phenotypic markers in a CSC subpopulation [164]. The only CSC-specific antigens that are used for CAR T cell therapy include CD133, can be located also in normal brain tissues, hematopoietic stem cells, endothelial progenitor cells, and ALDH which is found in hematopoietic stem or progenitor cells [165,166].

In order to resolve the aforementioned toxicity concern, significant measures ought to be applied prior to ACT initiation to enhance the selective activity of CSC-targeted CAR T cells against tumors. For instance, CAR T cell design can be carried out in such a way that the resulting cells selectively target TAAs instead of normal tissue, while ensuring that CAR T cells are eliminated in vivo after the initiation of extreme and uncontrollable AEs. The first can be achieved through CAR T cells which simultaneously target two TAAs, while the latter can be achieved through TAAs encoded with suicide genes, in order to eliminate CAR T cells in case of AEs [167,168]. Several studies have proposed the use of a small molecule as a “switch” that indirectly monitors CAR T cell activity [169,170]. To date, off-target toxicity is resolved through the administration of targeted immunosuppressive agents, such as tocilizumab (an anti-IL-6-antibody) or steroid therapy, aiming to better regulate cytokine release syndrome (CRS) [171]. While there has been sufficient knowledge that accumulated on BCSC properties, the majority of results originate from investigations involving xenografts of immuno-compromised nude mice. Hopefully, upcoming studies will investigate humanized mice and immunodeficient strains with engrafted immune systems that possess human-like characteristics [172].

### 5.2. Oncolytic Virotherapy (OVT)

Regarding the GLV-1h153 Lister strain, clinical evidence has demonstrated the safety and relative lack of toxicity (<1% toxicity rate) related to the vaccine administration. Its safety can be assured based on the historical evidence on the use of vaccines, such as that of Edward Jenner used against the spread of smallpox, while the lack of substantial toxicity can be attributed to the large genome of the virus, which allows for large-scale alterations in its genetic code and thus facilitates tumor-specific targeting [143]. In the case of combination therapy with the vvDD strain, its toleration rate was favorable as it had reached a maximum feasible dose of 3 × 109 pfu. A lack of major DLT, with the exception of one instance of treatment-related severe AEs, allowed for dose escalation. In that incident, the patient developed grade 3 rib pain (3 × 107 pfu dose), with no radiologic evidence of damage to the lungs or evidence of infection in the rib, which required admission to a rib hospital 7 days after injection. Besides that, there is evidence of tumor-targeted activity without the infection of otherwise healthy physiological tissue or systemic toxicity. In order to fully determine the use of OVT, the FDA requires both an intratumoral as well as intravenous trial in a timely manner. As of today, accumulated evidence supports the use of OVT without significant AEs.

### 5.3. DC-Based Vaccines

There is a limited number of clinical trials which investigate the toxicity of DC-vaccines targeting BCSCs. In vivo studies in humanized mice models, vaccinated with 10^6 cells/mice and transplanted with HSCs, showed fluctuations in body weight, WBCs, and human leukocytes in murine peripheral blood, compared to the controlled mice. Specifically, the weight of treated mice significantly increased within the first few weeks and gradually decreased, particularly in mice that eventually died [149]. Overall, DC-based vaccines used for the treatment of BC displayed a favorable toxicity profile. According to Santisteban et al. (2021), the inclusion of DC vaccines with NAC in HER2-negative BC patients (NCT01431196) is considered a safe approach, as it enhances the pCR rate, especially amongst PD-L1-negative tumors [173]. Additionally, they included patients suffering from HER2-negative BC, with or without immunotherapy based on DC vaccination. The outcomes of these studies propose that the DC vaccine-based immunotherapeutic intervention has a significant effect on the decrease in tumor size of BC.

Moreover, the implementation of cancer testis antigens (CTAs), which belong to the group of TAAs, shows encouraging results in HER2+ BC. MAGE-A and NY-ESO-1 are the main CTAs being tested in ongoing phase I clinical trials. Notably, the vaccination of five peptides (CDCA1, URLC10, KIF20A, DEPDC1, and MPHOSPH1) in nine metastatic and advanced BC patients led to immunization locally and systemically in 44% and 78% of patients. In fact, 60% of patients who completed two vaccination cycles showed tumor regression. Further clinical trials are required, which will focus on the immunogenic effect of BCSCs, while determining toxicity [174].

### 5.4. NK Cells

NK cells possess effective antibody-dependent cellular cytotoxicity properties, bypassing the systemic toxicity that is expected during a systemic NK cell stimulation, as commonly noted during a systemic IL-2 or IL-12 therapy [175,176]. Although NK cell-targeted immunotherapy has demonstrated significant effectiveness when the immunotherapeutic course is combined with radiotherapy, it has yet to be determined whether NK transfer, especially a chronic one that aims to maximize therapeutic effects, could lead to the development of resistance on the behalf of CSCs. Wang et al. (2014) suggest that primary BCSCs that exhibit increased ALDH activity possess the capability of evading NK cell-induced cell death through the downregulation of NKG2D ligand presence on the membrane. However, they do not take into consideration the synergistic effect RT, which downregulates NKG2D [102]. Another issue presented involves the efficacy of NK cell treatment. Gennari et al. (2004) observed antibody-dependent lytic activity from isolated PBMCs, compared with that of nonresponders [177]. For that cause, they proposed NK cell treatment combined with a tumor-targeting antibody that induces immunogenic response via the patient’s innate immune system, in order to enhance the intervention against BC and HER2-expressing tumors. It is clear that further investigation is required to clarify the range of effect and efficacy of chronic treatments based on either NK cells, or RT on the downregulation of NKG2D ligands.

## 6. Discussion

The current state of BC immunotherapy, along with the latest advancements in the field, seem promising for BC patients and their respective physicians. However, there is not enough space for additional research to be conducted. Through the examination of the implicated biochemical pathways and pathophysiology of BCSCs, as well as the retrospective evaluation of already applied interventions, there is hope for potential developments in the combat against BC.

## 7. Conclusions

Considering the aforementioned, CSCs have a crucial role in the occurrence, survival, and progression of neoplastic diseases. Especially in the case of BC, they play a crucial role in determining the disease profile and selection of the optimal therapeutic strategy. Granted the significant milestones that immunotherapy has achieved during recent decades, the exploitation of altered molecular and pathophysiological characteristics poses a valid point during the design and implementation of immunotherapeutic interventions. The assessment of novel and existing approaches can lead the way to a future of more targeted and efficient therapies.

## Figures and Tables

**Table 1:** Summary of immunotherapeutic strategies, their intervention, and therapeutic effect.

Immunotherapy	Intervention	Therapeutic Effect
Cytokine-induced killer (CIK) cells, NK cells, CD8+ T cells, and γδ T-cells	Targeting CSCs	Ideal for BCSCs [90]; promotes specific elimination [91]
DC-based vaccines	Targeting CSCs	Specifically targets BCSCs [93]; promotes specific elimination
Adoptive T-cell therapy	TIL isolation, culturing, and reinfusion	Enhances antitumor immunity through T-cell activation [92]
Oncolytic virotherapy (OVT)	Immunogenic cell death and T-cell activation	Induces antitumor immunity via immunogenic cell death [92]
Combination with other immunotherapies	Various immunotherapies combined	Synergistic effect utilizing oncolytic viruses, DC-based vaccines, and checkpoint blockades
